# Antioxidant Supplementation Hinders the Role of Exercise Training as a Natural Activator of SIRT1

**DOI:** 10.3390/nu14102092

**Published:** 2022-05-17

**Authors:** Carmine Sellitto, Graziamaria Corbi, Berenice Stefanelli, Valentina Manzo, Marta Trucillo, Bruno Charlier, Francesca Mensitieri, Viviana Izzo, Angela Lucariello, Angelica Perna, Germano Guerra, Antonio De Luca, Amelia Filippelli, Valeria Conti

**Affiliations:** 1 Department of Medicine, Surgery and Dentistry “Scuola Medica Salernitana”, Medicina Traslazionale dello Sviluppo e dell’Invecchiamento Attivo, University of Salerno, Via S. Allende, 84081 Baronissi, Italy; csellitto@unisa.it (C.S.); bcharlier@unisa.it (B.C.); 2 Clinical Pharmacology Unit, San Giovanni di Dio e Ruggi d’Aragona University Hospital, Via San Leonardo 1, 84131 Salerno, Italy; vmanzo@unisa.it (V.M.); afilippelli@unisa.it (A.F.); vconti@unisa.it (V.C.); 3 Department of Medicine and Health Sciences, University of Molise, 86100 Campobasso, Italy; angelica.perna@unimol.it (A.P.); germano.guerra@unimol.it (G.G.); 4 Postgraduate School of Clinical Pharmacology and Toxicology, University of Salerno, Via S. Allende, 84081 Baronissi, Italy; bstefanelli@unisa.it; 5 Department of Mental and Physical Health and Preventive Medicine, Section of Human Anatomy, University of Campania “Luigi Vanvitelli”, 80138 Naples, Italy; marta-trucillo@libero.it (M.T.); antonio.deluca@unicampania.it (A.D.L.); 6 Department of Medicine, Surgery and Dentistry “Scuola Medica Salernitana”, University of Salerno, Via S. Allende, 84081 Baronissi, Italy; fmensitieri@unisa.it (F.M.); vizzo@unisa.it (V.I.); 7 Department of Sport Sciences and Wellness, Section of Human Anatomy, University of Naples “Parthenope”, 80100 Naples, Italy; angela.lucariello@uniparthenope.it

**Keywords:** endurance training, sirtuins, vitamins, athletes, antioxidant capacity

## Abstract

Exercise training (ET) is a natural activator of silent mating type information regulation 2 homolog 1 (SIRT1), a stress-sensor able to increase the endogenous antioxidant system. SIRT1 activators include polyphenols and vitamins, the antioxidant properties of which are well-known. Antioxidant supplements are used to improve athletic performance. However, they might blunt ET-related benefits. Middle-distance runners (MDR) taking (MDR-S) or not taking antioxidant supplements (MDR-NoS) were compared with each other and with sedentary subjects (CTR) to evaluate the ET effects on SIRT1 levels and oxidative stress, and to investigate whether an exogenous source of antioxidants could interfere with such effects. Thirty-two MDR and 14 CTR were enrolled. MDR-S took 240 mg vitamin C and 15 mg vitamin E together with mineral salts. SIRT1 mRNA and activity were measured in PBMCs. Total oxidative status (TOS) and total antioxidant capacity (TEAC) were determined in plasma. MDR showed higher levels of SIRT1 mRNA (*p* = 0.0387) and activity (*p* = 0.0055) than did CTR. MDR-NoS also showed higher levels than did MDR-S without reaching statistical significance. SIRT1 activity was higher (*p* = 0.0012) in MDR-NoS (1909 ± 626) than in MDR-S (1276 ± 474). TOS did not differ among the groups, while MDR showed higher TEAC levels than did CTR (2866 ± 581 vs. 2082 ± 560, *p* = 0.0001) as did MDR-S (2784 ± 643) and MDR-NoS (2919 ± 551) (MDR-S vs. CTR, *p* = 0.0007 and MDR-NoS vs. CTR, *p* = 0.003). TEAC (β = 0.4488356, 95% CI 0.2074645 0.6902067; *p* < 0.0001) and the MDR-NoS group (β = 744.6433, 95% CI 169.9954 1319.291; *p*= 0.012) predicted SIRT1 activity levels. Antioxidant supplementation seems to hinder the role of ET as a natural activator of SIRT1.

## 1. Introduction

A sedentary lifestyle is one of the most important risk factors for clinical conditions associated with oxidative stress, such as type 2 diabetes and cardiovascular diseases (CVD) [1,2,3]. Exercise training (ET) is recommended as a valuable therapeutic approach by the International Health Authorities, as it provides health benefits to patients belonging to several clinical settings [4,5]. ET contrasts oxidative stress by decreasing radical oxygen species (ROS) and other oxidant molecules and/or increasing antioxidant ones [6]. On the other hand, because of increased oxygen consumption, ROS produced during ET may overcome the capacity of the endogenous antioxidant system to detoxify them [7,8]. Nowadays, it has been clarified that ROS are signaling molecules that, triggering an adaptive antioxidant response, work to maintain redox homeostasis [9]. This underpins the concept of mitohormesis [10]. ET is a natural activator of Sirtuin 1 (SIRT1), which is an NAD^+^-dependent deacetylase acknowledged as a life- and health span-prolonging agent [11,12,13,14]. SIRT1, activated during ET, can counteract aging and age-associated diseases by increasing the cellular antioxidant capacity and improving mitochondrial biogenesis [13,14,15]. However, the ET-related effects, including SIRT1 activation, strongly depend on the type, intensity, and duration of the training [16,17,18,19]. In particular, aerobic ET has been shown to elicit the optimal modulation of SIRT1 activity under oxidative stress injuries and anti-senescent and antioxidant effects in comparison to mixed (aerobic/anaerobic) and anaerobic ET [16]. However, these beneficial effects can be eliminated when aerobic training is performed at a greater workload [17].

Other natural activators of SIRT1 include several phenolic plant extracts and vitamins whose antioxidant properties are widely acknowledged [20,21]. Antioxidants can contribute to preventing or contrasting oxidative stress and its associated cellular damage. Indeed, supplements, especially those containing vitamins and other micronutrients, are commonly used to improve athletes’ wellness and performance [22]. Despite this, the effects of antioxidant supplementation have not yet been elucidated, particularly in athletes performing endurance training [23].

In this study, we compared the effects on SIRT1 and oxidative stress markers in endurance athletes using or not antioxidant supplements to investigate whether an exogenous source of antioxidants could interfere with the ET-related outcomes.

## 2. Materials and Methods

### 2.1. Study Design

This was an observational study in which athletes practicing middle distance running (MDR) and age-matched sedentary volunteers (CTR) were enrolled. Inclusion criteria were age ≥18 years, MDR performed for at least 6 months, certificate of sport suitability, and signed informed consent before initiation of the study. Exclusion criteria were acute or chronic diseases, severe injuries in the last 6 months, and the use of medicines.

All athletes were asked to report information on their dietary habits and use of supplements, tobacco and alcohol consumption, and the frequency and workload of training sessions. Fifteen days before blood collection, all athletes received a food frequency questionnaire.

### 2.2. Study Population

All MDR belonged to an amateur sports association called “Atletica Salerno”. Taking into account what was declared in their reports, they were divided into two groups. One of them (MDR-S) consumed, every training day and in conjunction with meals, antioxidant supplementation (S) consisting of 240 mg vitamin C and 15 mg vitamin E, together with 861 mg sodium, 555 mg chlorine, 381 mg potassium, and 66 mg magnesium. The other group did not use any antioxidant supplementation (MDR-NoS). Dietary indications were given to the athletes one week before blood sampling to reflect the similar distribution of carbohydrates, lipids, proteins, and fluids. Particular care was given to ensure that there were no significant differences between the two groups in the dietary habits and other factors that could influence serum antioxidant status. All participants signed informed consent, and the study received approval from the Campania Sud Ethics committee (Observational Study no. 86/2020).

### 2.3. Blood Sample Collection

Blood samples were drawn from each participant and collected in the fasting condition in BD Vacutainer^®^ Blood Collection Tubes (PA-USA). All MDR had refrained from training for 7 days. Peripheral blood mononuclear cells (PBMCs) were isolated by Ficoll–Paque PLUS (GE Healthcare, Munich, Germany), according to the manufacturer’s procedures. Serum samples were obtained by centrifugation at 1500× *g* for 10 min. Aliquots of serum and PBMCs were frozen at −80 °C until analysis.

### 2.4. Measure of SIRT1 mRNA Levels

RNA was extracted from PBMCs using Trizol reagent (GeneAll Biotechnology Ltd., Seoul, Korea) and reversely transcribed into cDNA using a commercial kit (Applied Biological Materials Inc.-ABM, Richmond, VA, Canada). Five microliters of cDNA were used in a total 20 µL PCR mix containing 12.5 µL of SYBR GREEN PCR Master Mix (Applied Biosystems™, MA, USA), 1.25 µL of each primer, and 5µL of water. The primer sets used to amplify SIRT1 cDNA were: forward 5′-CCC TCA AAG TAA GAC CAG TAG CA-3′ and reverse 5′-AGT CTC CAA GAA GCT CTA CAT CA-3′. Actin was denoted as the internal control. The actin primers were: forward 5′-CAA CCG CGA GAA GAT GAC CC-3′ and reverse 5′-GAG GCG TAC AGG GAT AGC AC-3′. Gene expression was quantified using QuantStudio 3 Real-Time PCR System Software (Applied Biosystems™, MA, USA). After initial denaturation for 10 min at 95 °C, the amplification was carried out for 40 cycles (95 °C for 5 s, 60 °C for 30 s). The experiments were performed in triplicate, and data were analyzed by using the 2−ΔΔC*t* method as relative quantification.

### 2.5. SIRT1 Activity

Nuclei were extracted from PBMCs using a commercial kit (EpiGentek Group Inc., New York, NY, USA). Then, SIRT1 activity was measured using a deacetylase fluorometric assay kit (Sir2 Assay Kit, CycLex, Ina, Nagano, Japan) according to the manufacturer’s procedures. Values were reported as relative fluorescence/μg of protein (AU). All data were expressed as mean ± SD of three independent experiments.

### 2.6. TOS Assay

A total oxidant status (TOS) assay evaluates peroxide concentrations in serum samples by measuring the oxidation of the ferrous ion in the presence of xylenol orange in an acidic environment. To minimize serum sample background noise and to quantify the level of free radicals, a modified method by Pavlatou et al. was used [24]. Ferrous-xylenol orange (FOX) reagent was prepared in 25 mM sulfuric acid (pH 1.75) by adding 8 mM ferrous sulfate and 2.5 mM xylenol orange. Ninety-six-well plates were filled with 45 μL of serum samples and 300 μL of 25 mM H_2_SO_4_, and absorbance was measured at 610 nm (T_0_). Then, 15 μL of FOX reagent was added to each sample, and, after 10 min of incubation at room temperature, the absorbance was measured at 610 nm (T_10_). Peroxide plasmatic concentration was measured as the ratio between samples and standards Abs according to the following equation:

$$\mathrm{peroxide}\;\mathrm{concentration}=\left(\frac{\Delta \mathrm{Abs}\;\mathrm{sample}}{\Delta \mathrm{Abs}\;\mathrm{standard}}\right)\times \mathrm{standard}\;\mathrm{concentration}$$

where

∆ABS = Abs T_10_–Abs T_0_

All data were expressed as mean ± SD of three independent experiments.

### 2.7. TEAC Assay

Trolox equivalent antioxidant capacity (TEAC) was measured by the 2,2′-azinobis- (3-ethylbenzothiazoline-6-sulphonate) (ABTS) assay [25]. ABTS (5.8 mM) was mixed with 2 mM ammonium persulfate (NH_4_)_2_S_2_O_8_ in 100 mM PBS (pH 7.4), generating ABTS+ radical, after overnight incubation. Phosphate buffer solution (PBS) was used to dilute the ABTS^+^ radical stock solution, obtaining an absorbance of 0.75 O.D. at 734 nm (working solution). Serum samples were diluted 1:5 with 240 μL of the ABTS+ working solution, and then 10 μL of samples were put into 96-well plates. After 2 min of mixing, a microplate reader (Thermo Fisher Scientific, MA, USA) was used to measure the absorbance referring to a Trolox standard curve. The absorbance was expressed as μM of Trolox equivalents. All data were expressed as mean ± SD of three independent experiments.

### 2.8. Oxidative Stress Index

The ratio of TOS to TEAC values was used to calculate the oxidative stress index (OSI) according to the following formula: TOS (µM H_2_O_2_ equivalent per liter)/TEAC (µM Trolox equivalent per liter).

### 2.9. Statistical Analysis

Continuous data were expressed as mean ± standard deviation (SD), while categorical variables were expressed as percentages (%). The results were expressed as the mean ± SD of three independent experiments. Differences between multiple groups were analyzed using ANOVA with the Bonferroni post hoc test. Variables that demonstrated statistical significance in a univariate model were then included in a multivariate analysis. Multivariate analyses were performed to assess correlations among variables. All tests were two-sided, and values of *p* < 0.05 were considered to be statistically significant. All analyses were carried out using Stata version 16.0 (Stata Corporation, College Station, TX, USA).

## 3. Results

Thirty-two middle distance runners (MDR) and 14 age-matched sedentary controls (CTR) represented the study population. MDR taking antioxidant supplementation were indicated as MDR-S (*n* = 18), while those belonging to MDR not taking supplements were referred to as MDR-NoS (*n* = 14). Table 1 reports the main characteristics of the enrolled subjects. The groups (MDR-S and MDR-NoS) were well-balanced regarding sex. No differences in age, smoking habits, alcohol use, or dietary habits were found between the two groups of athletes and between athletes and CTR. CTR had a Body Mass Index (BMI) higher than both MDR-S and MDR-NoS. Neither training time/week nor training frequency/week differed between MDR-S and MDR-NoS.

### 3.1. SIRT1 mRNA Expression and Activity

**Figure 1:**
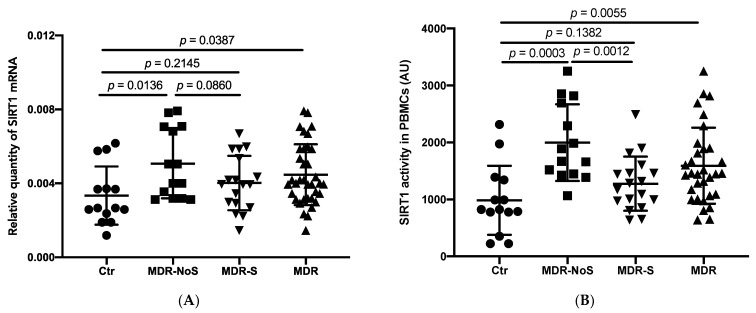
SIRTUIN 1 (SIRT1) mRNA expression (**A**) and activity (**B**) were measured in peripheral blood mononuclear cells (PBMCs) extracted from sedentary controls (CTR), middle distance runners not taking antioxidant supplements (MDR-NoS), and MDR taking antioxidant supplements (MDR-S). MDR indicates all the MDR, irrespective of antioxidants supplementation. CTR, MDR-NoS, MDR-S, and MDR are respectively indicated with a black circle, black square, black triangle with the vertex at the bottom, and black triangle with the vertex at the top. All data are expressed as mean ± SD.

As shown in Figure 1A, the MDR group demonstrated higher levels of SIRT1 mRNA compared with the CTR group (*p* = 0.0387). Notably, MDR-NoS showed higher levels than CTR (*p* = 0.0136), while there were no statistically significant differences between MDR-S and CTR or between MDR-S and MDR-NoS.

Similar to SIRT1 mRNA, MDR showed higher levels of SIRT1 activity compared with the CTR (*p* = 0.0055). MDR-NoS had the highest value, significantly higher compared both with CTR (*p* = 0.0003) and MDR-S (*p* = 0.0012). SIRT1 activity of MDR-S was higher when compared with CTR without reaching statistical significance (Figure 1B).

### 3.2. Oxidative Stress Markers

**Figure 2:**
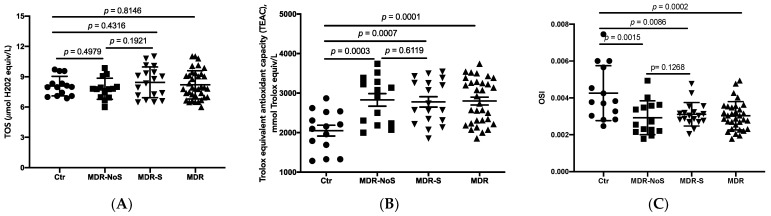
Total oxidative status (TOS) (**A**), Trolox equivalent antioxidant capacity (TEAC) (**B**), and oxidative stress index (TOS/TEAC, OSI) (**C**) were determined in the serum extracted from sedentary controls (CTR), middle distance runners not taking antioxidant supplements (MDR-NoS) and MDR taking antioxidant supplements (MDR-S). MDR indicates all the MDR irrespective of antioxidant supplementation. CTR, MDR-NoS, MDR-S, and MDR are respectively indicated with a black circle, black square, black triangle with the vertex at the bottom, and black triangle with the vertex at the top. All data are expressed as mean ± SD.

As shown in Figure 2A, no differences in TOS levels were found among the groups. Conversely, the MDR showed higher TEAC levels than did the CTR (*p* = 0.0001). Notably, both the MDR-S and MDR-NoS showed higher TEAC levels than did the CTR (MDR-NoS vs. CTR, *p* = 0.0003 and MDR-S vs. CTR, *p* = 0.0007). There were no statistically significant differences between MDR-S and MDR-NoS (Figure 2B).

Regarding OSI (TOS/TEAC), the CTR demonstrated the highest levels compared with the other groups. No differences were found between MDR-S and MDR-NoS (Figure 2C).

### 3.3. Linear Regression Analyses

**Figure 3:**
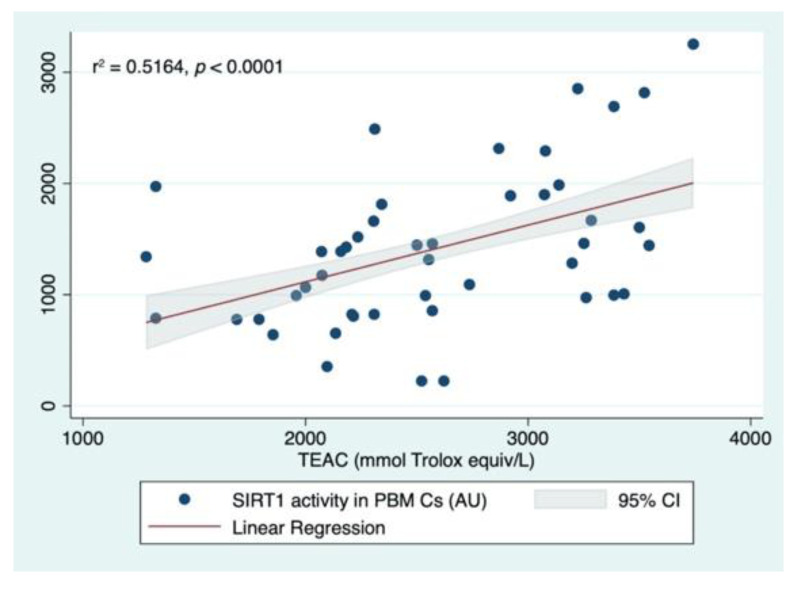
Linear regression analyses with SIRT1 (SIRT1) activity measured in peripheral blood mononuclear cells (PBMCs) and Trolox equivalent antioxidant capacity (TEAC) measured in serum.

A multiple linear regression analysis was performed to test the predictors of SIRT1 activity levels. The analysis tested if age, sex (female), BMI, TEAC, TOS levels, and Ctr, MDR-NoS, or MDR-S groups significantly predicted SIRT1 levels. The fitted regression model was −124.064–0.143 * (Age) −97.546 * (Women) +17.021 * (BMI) +0.449 * (TEAC) −27.973 * (TOS) +744.643 * (MDR-NoS) +61.879 * (MDR-S) +360.588 (MDR total). The overall regression was statistically significant (r^2^ = 0.3725, F (8,69), *p* = 0.0001). It was found that TEAC (β = 0.449, 95% CI 0.208 0.690; *p* < 0.0001) and the MDR-NoS group (β = 744.643, 95% CI 169.995 1319.291; *p* = 0.012) significantly predicted SIRT1 activity levels (Figure 3).

**Figure 4:**
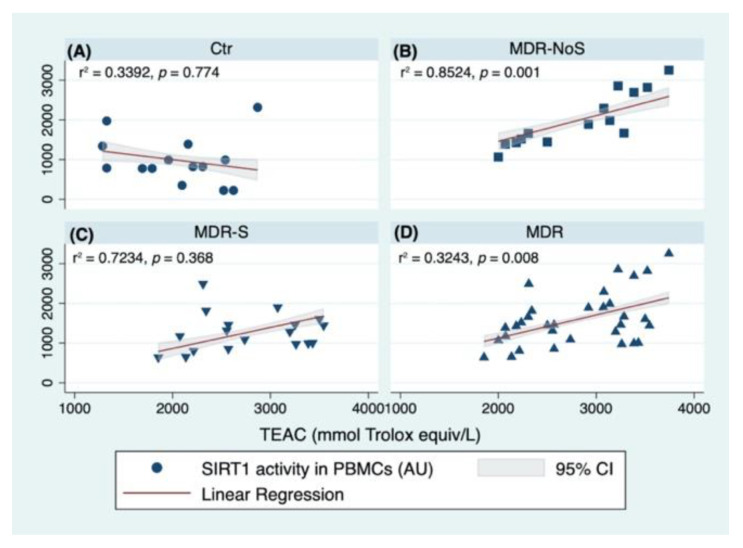
Linear regression analyses with SIRT1 (SIRT1) activity measured in peripheral blood mononuclear cells (PBMCs) and Trolox equivalent antioxidant capacity (TEAC) measured in serum of CTR (**A**), MDR-NoS (**B**), MDR-S (**C**), and MDR (**D**). The groups were: sedentary controls (CTR), middle distance runners (MDR) not taking antioxidant supplements (MDR-NoS), and MDR taking antioxidant supplements (MDR-S), indicated, respectively, with a black circle, black square, black triangle with the vertex at the bottom, and black triangle with the vertex at the top. All data are expressed as mean ± SD. All data are expressed as mean ± SD.

Then, another linear regression analysis was performed to test the predictors of SIRT1 activity levels in each of the considered groups. The analysis tested if age, sex (female), BMI, TEAC, and TOS levels significantly predicted SIRT1 activity levels by the groups. In the Ctr group, the fitted regression model was: 1982.697–27,6105 * (Age) −545.369 * (Women) +57.987 * (BMI) −0.3090395 * (TEAC) −45.922 * (TOS). The overall regression was not statistically significant (r^2^ = 0.314, F (5,8), *p* = 0.619), and no significant predictors were identified (Figure 4A). In the MDR-NoS, the fitted regression model was: −2168.175 + 9.648 * (Age) + 64.215 * (Women) +44.258 * (BMI) +1.031 * (TEAC) −29.290 * (TOS). The overall regression was statistically significant (r^2^ = 0.830, F (5,8), *p* = 0.006). The only significant predictors of the SIRT1 activity levels in the MDR-NoS group were the TEAC levels (β = 1.031, 95% CI 0.489 1.574; *p* = 0.002) (Figure 4B). In the MDR-S group, the fitted regression model was: −276.417 + 16.558 * (Age) −276.417 * (Women) +11.239 * (BMI) −0.139 * (TEAC) +121.103 * (TOS). The overall regression was not statistically significant (r^2^ = 0.422, F (5,12), *p* = 0.198), and no significant predictors were identified (Figure 4C). Finally, when considering the MDR-NoS and the MDR-S groups together (MDR), the overall regression was not statistically significant (r^2^ = 0.273, F (5,26), *p* = 0.120). The only significant predictors of the SIRT1 activity levels were the TEAC levels (β = 0.603, 95% CI 0.185 1.021; *p* = 0.006), likely due to the greatly significant association of SIRT1 activity and TEAC levels found in the MDR-NoS group (Figure 4D).

**Figure 5:**
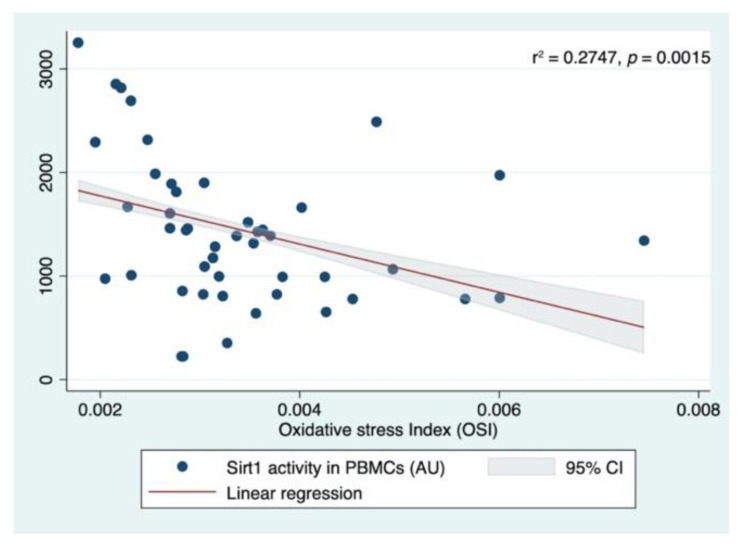
Linear regression analyses with SIRT1 (SIRT1) activity measured in peripheral blood mononuclear cells (PBMCs) and Oxidative Stress Index (OSI) calculated as ratio of TOS on TEAC.

Because the OSI index is considered a more reliable parameter of oxidative stress, another multivariate analysis was performed, changing the TEAC and TOS with OSI as a possible predictor. The analysis tested if age, sex (female), BMI, OSI levels, and Ctr, MDR-NoS, or MDR-S groups significantly predicted the SIRT1 levels. The fitted regression model was 1843.672 + 0.1891939 * (Age) −138.0873 * (Women) −5.711977 * (BMI) −149499.1 * (OSI) +778.9953 * (MDR-NoS) +85.39653 * (MDR-S) +388.846 (MDR). The overall regression was statistically significant (r^2^ = 0.2747, F (7,70), *p* = 0.0015). It was found that only participation in the MDR-NoS group (β =778.9953, 95% CI 212.1037 1345.8871; *p* = 0.008) significantly predicted the SIRT1 activity levels (Figure 5).

**Figure 6:**
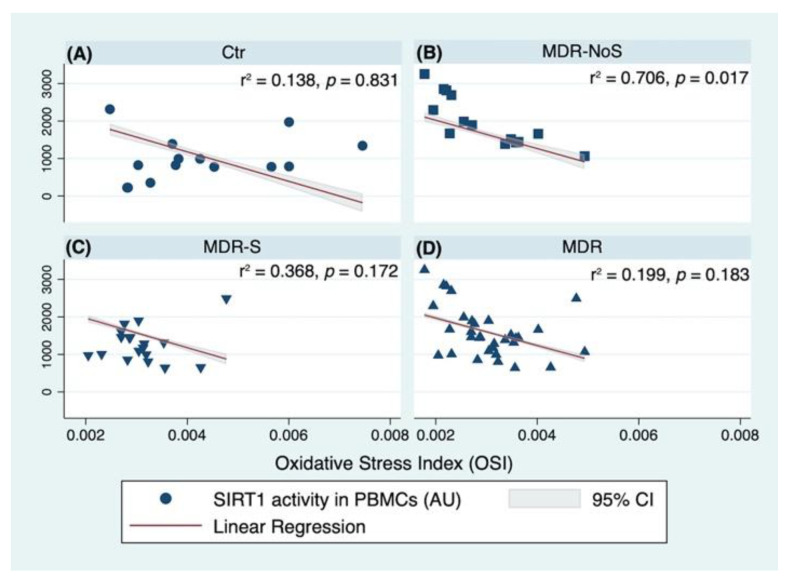
Linear regression analyses with SIRT1 (SIRT1) activity measured in peripheral blood mononuclear cells (PBMCs) and Oxidative Stress Index (OSI) calculated as the ratio of TOS on TEAC, CTR (**A**), MDR-NoS (**B**), MDR-S (**C**), and MDR (**D**). The groups were: sedentary controls (CTR), middle distance runners (MDR) not taking antioxidant supplements (MDR-NoS), and MDR taking antioxidant supplements (MDR-S), indicated, respectively, with a black circle, black square, black triangle with the vertex at the bottom, and black triangle with the vertex at the top. All data are expressed as mean ± SD. All data are expressed as mean ± SD.

Another analysis tested if age, sex (female), BMI, and OSI significantly predicted SIRT1 levels by the groups. In the Ctr group, the fitted regression model was: 1117.95–10.955 * (Age) −294.074 * (Women) +5.265 * (BMI) +10,3207 * (OSI). The overall regression was not statistically significant (r^2^ = 0.138, F (4,9), *p* = 0.831), and no significant predictors were identified (Figure 6A). In the MDR-NoS, the fitted regression model was: 3033.987–1.495 * (Age) +199.623 * (Women) +28.983 * (BMI) −586,680.4 * (OSI). The overall regression was statistically significant (r^2^ = 0.706, F (4,9), *p* = 0.017). The only significant predictors of the SIRT1 activity levels in the MDR-NoS group was the OSI (β = −586,680.4, 95% CI -895,308–278,052.8; *p* = 0.002) (Figure 6B). In the MDR-S group, the fitted regression model was: −65.663 + 22.852 * (Age) −317.383 * (Women) −11.786 * (BMI) +132,664.8 * (OSI). The overall regression was not statistically significant (r^2^ = 0.368, F (4,13), *p* = 0.172), and no significant predictors were identified (Figure 6C). Finally, when considering the MDR-NoS and the MDR-S groups together (MDR), the overall regression was not statistically significant (r^2^ = 0.199, F (4,27), *p* = 0.183). The only significant predictors of the SIRT1 activity levels was the OSI (β = −385,027.6, 95% CI −706,308.1 – 63,747.09; *p*= 0.021), likely due to the greatly significant association of SIRT1 activity and OSI found in the MDR-NoS group (Figure 6D).

## 4. Discussion

The main finding of this study is that aerobic ET combined with an exogenous source of antioxidants is associated with an increase in systemic antioxidant capacity but not in SIRT1 activity. When a multiple linear regression analysis was performed, the best predictors of SIRT1 activity levels were the TEAC (*p* < 0.0001, Figure 3), and participation in the MDR-NoS group (r^2^ = 0.3725, *p* = 0.012). This finding was confirmed by a second linear regression analysis performed by groups. In fact, in the MDR-NoS, TEAC levels were the only significant predictors of the SIRT1 activity levels, while no predictors were identified in Ctr and MDR-S. These findings suggest that the ET alone induced an increase of antioxidant molecules, and this increase was associated with augmented SIRT1 activity levels. On the other hand, when supplementation was added to the ET, these beneficial effects were lost.

The beneficial effects of ET (mainly aerobic) have been linked to its capacity for triggering an antioxidant response through a transient and moderate increase of ROS based on the concept of mitohormesis [8]. On the other hand, an accumulation of oxidative stress during ET can lead to damage of all cellular constituents and could impair athletic performance [26]. For this reason, athletes often resort to antioxidant supplementation, including vitamins E and C, to prevent or reduce possible ET-related negative effects, especially muscle damage [26,27].

ET is a natural activator of SIRT1, which, acting in turn as a stress-sensor, can mount an adaptive response to a variety of stressors, including ROS produced by increased oxygen consumption [14,28]. However, this effect strongly varies based on the type and intensity of the ET [16,17,29].

An exogenous source of antioxidants could interfere with ET-related outcomes, leading to effects that are actually detrimental rather than beneficial. Ristow et al. demonstrated that oral supplementation of 500 mg vitamin C twice a day and 400 IU vitamin E once a day prevented the ET-related health-promoting effects, including the enhancement of endogenous responses to ROS [30]. These authors concluded that antioxidant supplementation should not be recommended in trained individuals [10].

In the present study, MDR-S, taking antioxidant supplements including an average dose of 240 mg vitamin C and 15 mg vitamin E daily, showed a similar amount of OSI (TOS/TEAC) of that measured in the MDR-NoS, who did not take supplements. Both of them showed low levels of OSI compared with sedentary subjects.

Several studies showed that increased levels of sirtuins in athletes compared with sedentary controls were associated with improved mitochondrial biogenesis and antioxidant function [14,31]. The aforementioned studies were carried out using muscle biopsies; therefore, they focused on ET-mediated local effects [31].

Similarly, the effects of daily intake of vitamins C and E were investigated using muscle samples [32]. Paulsen et al., in a double-blind randomized controlled trial, observed that ET-related muscle adaptations were hindered in athletes taking 1000 mg of vitamin C and 235 mg of vitamin E daily for 11 weeks. In particular, the intake of the vitamins led to an attenuation of the ET-related increases in markers of mitochondrial biogenesis. The authors concluded that, although this finding was not associated with loss of athletic performance, caution should be taken when considering combining antioxidant supplementation with endurance exercise [32].

Roberts et al. showed that supplementation of 1000 mg of vitamin C did not impair training-induced improvements in exercise performance in recreationally active males who performed high-intensity interval running training [33]. On the other hand, Dutra et al. reported that a reduction in muscle performance was observed in healthy women after ten weeks of twice-weekly strength training and administration of vitamins E (400 IU/day) and C (1000 mg/day) [34].

Other studies did not find interference by the antioxidant supplements on the ET-related effects. Theodorou et al. observed that neither muscle performance nor blood redox status was influenced by the administration of vitamins E (400 IU/day) and C (1000 mg/day) in a group of healthy volunteers participating in a program of eccentric ET twice weekly for four weeks [35].

A review including crossover and controlled trials highlighted mixed results. Some studies reported that vitamin C at a dosage of more than 1 g daily could impair physical performance through a possible reduction in mitochondrial biogenesis, while others did not observe a statistically significant impact [36].

It has been suggested that a well-balanced diet including vitamins could act synergistically with a regular ET to optimize, rather than contrast, its antioxidant properties [36]. The type and intensity of ET, as well as antioxidant supplement dosages, can influence the results of different studies.

Notably, all the athletes enrolled in the present investigation performed the same type of ET and took 240 mg of vitamin C and 15 mg of vitamin E daily. Such homogeneity allowed us to better investigate the effects of an endurance ET (i.e., MDR) combined with antioxidant supplementation.

A possible limitation could be the lack of a control group, including sedentary individuals taking the same antioxidant supplements used by the athletes. However, the present is an observational study, and the inclusion of this group would have implied the need to prescribe these supplements. Moreover, further studies should be planned to assess separately the effects exerted by vitamin C or vitamin E on the ET-related outcomes.

## 5. Conclusions

Consistent with the concept of mitohormesis, it is conceivable that in MDR-S, the presence of an exogenous source of antioxidants did not justify activation of SIRT1 to stimulate the endogenous antioxidant system like that observed in MDR-NoS.

Antioxidant supplementation based on vitamin C and E, although at a low dosage, could interfere with the ET-related effects on oxidative redox state and SIRT1 activity.

Personalized antioxidant interventions, based on the identification of possible antioxidant deficiencies, could enhance the beneficial effects of ET without interfering with ET-related adaptive responses.

## Figures and Tables

**Table 1:** The main characteristics of the study population.

	MDR (*n* = 32)	MDR-S (*n*= 18)	MDR-NoS (*n*= 14)	CTR (*n* = 14)	*p*-Value
Age, yr. (mean ± SD)	50.69 ± 7.06	53.5 ± 8.04	47.07 ± 10.32	46.86 ± 8.7	0.072
Sex (F/M), *n*	16/16	9/9	7/7	9/5	0.816
BMI, kg/m^2^ (mean ± SD)	22.505 ± 2.08	22.575 ± 2.10	22.415 ± 2.14	24.77 ± 2.02	0.006 *
Smokers, *n* (%)	3 (9.38)	1 (5.56)	2 (14.29)	1 (7.14)	0.848
Former smokers, *n* (%)	12 (37.50)	7 (38.89)	5 (35.71)	4 (28.57)	0.933
No smokers, *n* (%)	17 (53.12)	10 (55.56)	7 (50.00)	9 (64.29)	0.879
Moderate alcohol users, *n* (%)	24 (75.00)	12 (66.67)	12 (85.71)	6 (42.86)	0.075
Weekly frequency of training, times/week (mean ± SD)	3.19 ± 1.03	3.17 ± 1.15	3.21 ± 0.89	---	0.636
Training time per week in minutes (mean ± SD)	233.38 ± 65.53	239.22 ± 62.81	225.86 ± 40.34	---	0.158

SD, Standard Deviation; * CTR vs. MDR-NoS, *p* = 0.023; CTR vs. MDR-S, *p* = 0.026; CTR vs. MDR, *p* = 0.007. ---, no data.

## Data Availability

Not applicable.
